# Variability in viscoelastic haemostatic assay in major haemorrhage protocols: A unified approach or mixed signals?

**DOI:** 10.1111/tme.70003

**Published:** 2025-07-23

**Authors:** Akmez Latona, James Winearls, Kate Hill, Michelle Spanevello, Biswadev Mitra

**Affiliations:** ^1^ Emergency Department Ipswich Hospital Ipswich Queensland Australia; ^2^ Statewide Anticoagulation Committee Queensland Health Brisbane Queensland Australia; ^3^ LifeFlight Retrieval Medicine Toowoomba Queensland Australia; ^4^ School of Public Health and Preventive Medicine Monash University Melbourne Victoria Australia; ^5^ The University of Queensland Faculty of Medicine Brisbane Queensland Australia; ^6^ Intensive Care Unit Gold Coast University Hospital God Coast Queensland Australia; ^7^ Department of Haematology Princess Alexandra Hospital Brisbane Queensland Australia; ^8^ Central Laboratory Pathology Queensland Brisbane Australia; ^9^ Department of Haematology Royal Brisbane and Women's Hospital Brisbane Australia; ^10^ 1 Expeditionary Health Squadron Royal Australian Air Force Amberley Queensland Australia; ^11^ Emergency & Trauma Centre The Alfred Hospital Melbourne Victoria Australia

**Keywords:** critical bleeding, major Haemorrhage protocol, patient blood management, rotational Thromboelastometry, Thromboelastography, viscoelastic Haemostatic assay

## Abstract

**Background and Objectives:**

Viscoelastic haemostatic assays (VHA) are part of patient blood management (PBM) for bleeding, associated with reduced transfusions. This study reviewed all major haemorrhage protocols (MHPs) using VHA in Queensland, Australia, and assessed variability.

**Methods:**

VHA platforms in Queensland Health include rotational thromboelastometry (ROTEM® Sigma) and thromboelastography (TEG 6 s). PBM guidelines were searched for VHA‐guided MHPs. Outcomes included viscoelastic thresholds and transfusion recommendations.

**Results:**

Nineteen hospitals used VHA: sixteen with ROTEM and three with TEG. Among hospitals with ROTEM, fibrinolysis was assessed first in 13 algorithms, primarily using FIBTEM flat‐line (*n* = 6) or ML >5% (*n* = 5). Fibrinogen thresholds were FIBTEM A5 <10 mm (*n* = 15) and <12 mm (*n* = 1). Platelet thresholds included EXTEM A5 <25 mm (*n* = 2) or EXTEM A5 <35 mm (*n* = 6) as isolated criteria, and EXTEM A5 <35 mm combined with FIBTEM A5 >10 mm (*n* = 9) as combined criterion. Coagulation factor thresholds were EXTEM CT >90 s (*n* = 13), EXTEM CT >80 s (*n* = 2) and INTEM CT ≥240 s (*n* = 1).

TEG algorithms used CFF MA/A10 <15 mm (*n* = 3), <10 mm and <5 mm (*n* = 1). Platelet thresholds: CRT MA <50 mm (*n* = 3), and <25 mm (*n* = 1). Coagulation factor thresholds: CK R >9 min (*n* = 2) and CKH R >10 min (*n* = 1). Fibrinolysis: CRT LY30 >2.2% (*n* = 3). Doses varied across all algorithms: cryoprecipitate (10–30 U), FC (3–6 g), platelet (1–2 U), fresh frozen plasma (1–4 U), and prothrombin complex concentrate (PCC) (5–50 U/kg).

**Conclusion:**

VHA‐guided MHP showed marked variation with inconsistent transfusion thresholds. For similar clot kinetics, dosing of blood products and haemostatic agents differed, particularly PCC. Patients with the same coagulopathy may receive different treatment across hospitals. Centralised standardisation could improve PBM consistency.

**SUMMARY**

- Hospitals adopt a unified VHA algorithm when incorporating VHA into hospital‐wide Major Haemorrhage Protocols (MHPs).
- Substantial variation in VHA‐guided algorithms was observed, including inconsistent transfusion thresholds.
- For same clot kinetics, dosing of blood products and haemostatic agents differed across hospitals.
- Patients with same type and degree of haemostatic disturbances face varying haemostatic management across different hospitals.
- Harmonising VHA algorithms may improve consistency in Patient Blood Management.

## 1 INTRODUCTION

Viscoelastic haemostatic assays (VHA) represent real‐time whole blood assessment of ex‐vivo coagulation, reflecting the interaction between plasma, platelets, and other blood cells involved in haemostasis.^1^ Tailored algorithms have been developed to meet the specific requirements of haemostatic resuscitation in trauma, postpartum haemorrhage (PPH), cardiovascular surgery, and liver transplantation.^2^, ^3^ VHA‐guided patient blood management (PBM) has been associated with lower transfusion requirements, complications, and healthcare costs.^4^ However, the integration of VHA into PBM requires comprehensive technical and interpretive training, alongside robust educational and logistical support.^5^

Health services develop and validate their own VHA algorithms for guiding major haemorrhage protocols (MHP), based on local resources, timing, and blood component availability. Although specific algorithms exist for specialised areas, hospitals adopt a unifying algorithm when incorporating VHA into hospital side MHPs to ensure cohesive practice across various units. Currently, there is no centralised oversight for harmonising VHA algorithms. While many PBM guidelines recommend VHA, the quality of evidence supporting its application remains low.^6^, ^7^ Consequently, Australia's national critical bleeding guidelines stopped short of recommending specific actions based on VHA.^8^ We hypothesised that there may be variability in VHA algorithms in MHPs.

The aim of this study was to review adult hospital‐wide MHP guidelines that incorporated VHA across all public hospitals in Queensland, Australia. The objective was to assess differences in clot kinetics thresholds for transfusion of blood components and haemostatic agents directed at coagulopathy. Describing the variability between VHA‐guided MHP algorithms may inform future efforts toward harmonisation. A key clinical consideration was whether a patient with the same type of coagulopathy would receive different haemostatic treatment when presenting to different hospitals.

## 2 METHODS

### 2.1 Setting

Queensland, Australia, with over 5 million people, delivers public healthcare through 16 Hospital and Health Services (HHS), managing 106 hospitals across metropolitan, regional, and rural areas. Queensland Health (QH) uses two VHAs—Rotational Thromboelastometry (ROTEM®; Werfen, Munich, Germany) and Thromboelastography (TEG®; Haemonetics S.A., Signy Centre, Switzerland). All hospitals use either ROTEM® sigma or TEG® 6 s platforms, with no inter‐hospital variability in devices, reagents or cartridges; all were provided with the same device manual and reference ranges from the manufacturer.

### 2.2 Data sources

A search of the Queensland Health Electronic Publishing System using VHA, ROTEM, TEG, MHP, and critical bleeding identified relevant VHA‐guided MHPs. To include all hospitals, blood management committees from all public hospitals were consulted and confirmed VHA‐guided MHP use.

Adult hospital‐wide MHP and critical bleeding pathways incorporating VHA were collated, including transfusion thresholds, viscoelastic targets, and transfusion recommendations. Minor threshold variations (≤ vs. < and ≥ vs. >) were excluded.

### 2.3 Data analysis

Algorithms were reviewed for variations in viscoelastic thresholds and transfusion recommendations. Outcomes were VHA thresholds that defined haemostatic dysfunctions and guided administration of antifibrinolytics, fibrinogen, platelet, and coagulation factor replacement. Where variability existed, threshold frequencies were reported as counts and percentages.

This study was approved by Metro South Human Research Ethics Committees (HREC/2024/QMS/115320).

## 3 RESULTS

Nineteen hospitals incorporated VHA into their MHP, with 16 using ROTEM and 3 using TEG. Eight were tertiary hospitals. VHA machines were in multiple locations in six hospitals and placed in ICU (*n* = 10), OT (*n* = 9), pathology (*n* = 7), and ED (*n* = 1). Live ROTEM test results were accessible on desktop applications in all critical care areas at the point of testing. VHA was used exclusively for critical bleeding, with no hospitals reporting its use for bleeding risk assessment or pre‐procedural haemostatic evaluation. Additional criteria included haemodynamic instability (*n* = 10), anticipated RBC transfusion (>3 units in 1 h [*n* = 4] or >4 units in 4 h [*n* = 5]), assessing fibrinogen reserve (*n* = 1), clinical or laboratory signs of coagulopathy in significant haemorrhage (*n* = 1), FAST‐positive trauma (*n* = 2), bleeding on CT (*n* = 2), lactate ≥4 mmol/L (*n* = 1), and ongoing bleeding ≥150 mL/min (*n* = 1). Table 1 summarises VHA parameters and haemostatic interventions across algorithms.

**TABLE 1:** Variations in ROTEM and TEG based thresholds and blood transfusion.

ROTEM (*n* = 16)				
		Coagulation threshold	Transfusion recommendations	Transfusion dose
Fibrinolysis	Early	F Flat Line	TXA TXA and FC TXA and FC or Cryo	FC: 1 g/25 kg, 3–4 g TXA: 1 g Cryoprecipitate: 1 U/5 kg, 5–10 U apheresis, 10 U
		F CT >600 s	TXA	
		E A5 <35 mm	TXA	
		F Flat Line and E A5 <35 mm	TXA and FC or Cryo TXA and FC	
		F CT >300 and E A5 <35 mm	TXA and FC	
		F CT >600 s and E A5 <35 mm	TXA and FC	
		E or F ML >5%	TXA	
		E ML >15%	TXA	
	Late	E ML >15%	TXA	
		E or F ML >5%	TXA	
Fibrinogen (FIBTEM assay)		F A5 <12 mm	Cryo	FC: 1 g/25 kg, 2 g, 3 g, 4 g Cryoprecipitate: 1 U/5 kg 10 U, 14 U, 16 U, 20 U, 10–20 U, 5–10 U apheresis TXA: 1 g
		F A5 <10 mm	Cryo FC or Cryo FC or Cryo and TXA	
		F A5 8–10 mm	Cryo	
		F A5 7–10 mm	FC or Cryo	
		F A5 <8 mm	FC or Cryo FC	
		F A5 5–7 mm	Cryo	
		F A5 <6 mm	FC or Cryo	
		F A5 2–4 mm	FC or Cryo	
		F Green line	FC	
Platelet FIBTEM and EXTEM assays		E A5 <35 mm	Platelet	Platelet: 1 U
		E A5 <35 mm and F A5 >10 mm	Platelet	
		E A5 <35 mm and F A5 >12 mm	Platelet	
		E A5 <25 mm and F A5 <10 mm	Platelet and fibrinogen	
Coagulation factor EXTEM, FIBTEM and INTEM assays		E CT >90s	FFP PCC FFP or PCC	FFP: 1–2 U 2–4 U PCC: 5–10 U/kg 10 U/kg 12.5 U/kg 25 U/kg
		E CT >90s and E A5 <35 mm	FFP	
		E CT >90 and F A5 >10	FFP FFP or PCC	
		E CT >80s and F A5 >10 mm	FFP or PCC	
		E CT >80s and F A5 >12 mm	FFP or PCC	
		E CT 80–140 s and F A5 ≤ 10 mm	Fibrinogen	
		E CT >140 s and F A5 ≤ 10 mm	FFP and fibrinogen	
		I CT >240 s	FFP or PCC	

TEG (*n* = 3)			
	Coagulation threshold	Transfusion recommendations	
Fibrinogen	CFF A10 or MA <15	FC 3 g	
	CFF A10 <15	FC 3 g or cryoprecipitate 1 U/10 kg	
	CFF MA <15	FC 2 g or cryoprecipitate 10 U	
	CFF MA <10	FC 4 g or cryoprecipitate 20 U	
	CFF MA <5	FC 4‐6 g or cryoprecipitate 20–30 U and 1 g TXA	
Platelet	CRT MA <50 mm	1 U platelet	
	CRT MA <25 mm	2 U platelet	
	CRT A10 <44 mm	1 U platelet	
Coagulation factor	CKH R >10 min	4 U FFP or PCC 10–15 U/kg	
	CKR >9 min	2–4 U FFP or PCC 25–50 U/kg	
Heparin effect	CKH R < CKR	Protamine: 1 mg/100 units heparin	
Fibrinolysis	CRT LY30 >2.2%	1 g TXA	

Abbreviations: A10, amplitude at 10 min; A10, amplitude at 10 min CFF, citrated functional fibrinogen; A5, amplitude at 5 min; CKH, kaolin‐heparinase assay; CRT, citrated rapid TEG; Cryo, cryoprecipitate; CT, clotting time; E, EXTEM; F, FIBTEM; FC, fibrinogen concentrate; FC, fibrinogen concentrate; I, INTEM; LY30, clot lysis at 30 min; MA, maximum amplitude; ML, maximum lysis; PCC, prothrombin complex concentrate; R, reaction time; ROTEM, rotational thromboelastometry; TEG, thromboelastogram; TXA, tranexamic acid; TXA, tranexamic acid.

### 3.1 ROTEM (n = 16)

#### 3.1.1 Assessment of hyperfibrinolysis

**FIGURE 1:**
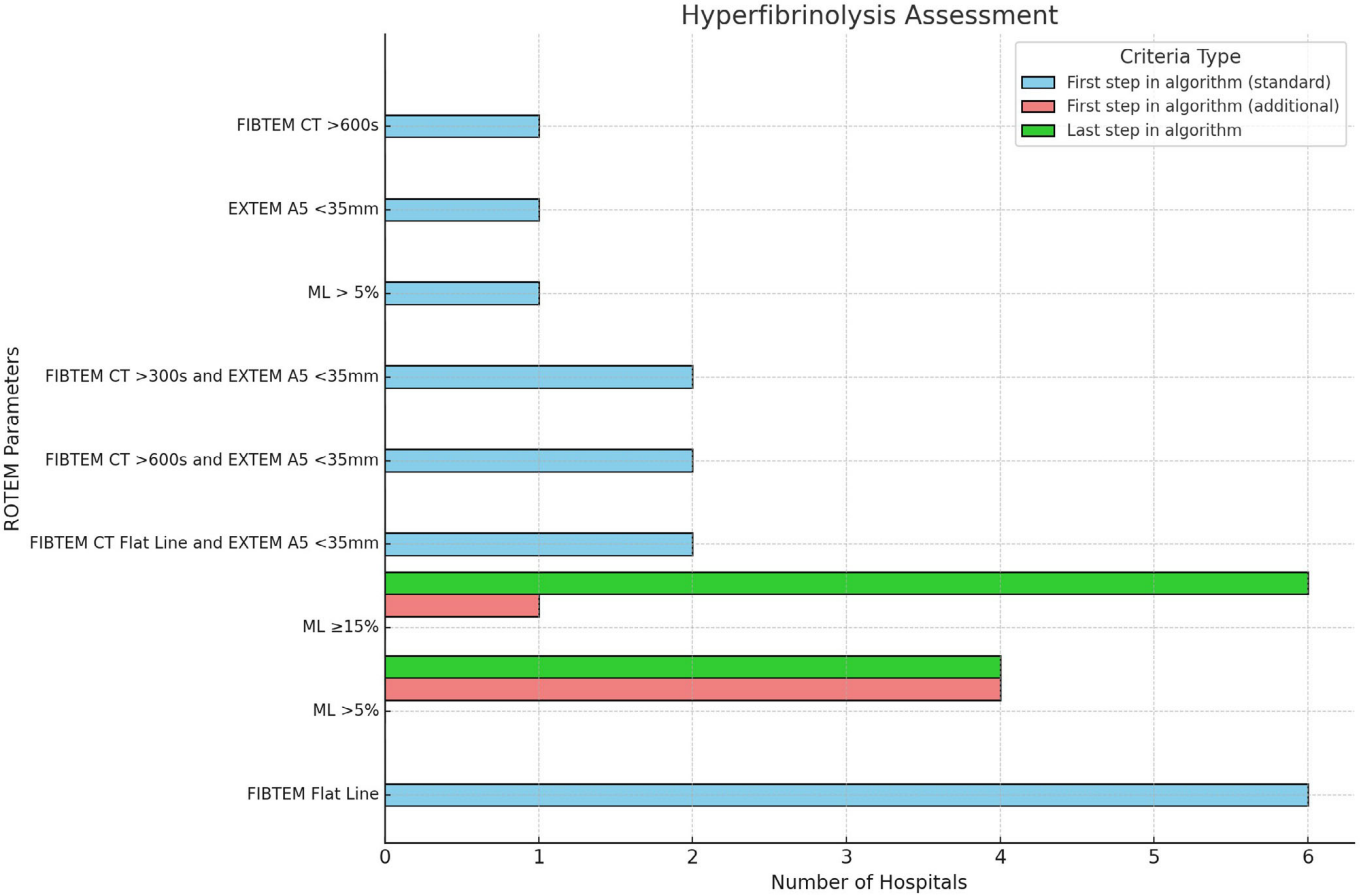
Diagnostic criteria for hyperfibrinolysis.

Of the 16 ROTEM algorithms, 13 (81%) included hyperfibrinolysis criteria in the first step, while 3 (19%) did not. The most common criteria were FIBTEM Flat Line (*n* = 6, 38%) and EXTEM ML >5% (*n* = 5, 31%) (Figure 1). Only one hospital used EXTEM ML >5% independently, while four (25%) incorporated it as an additional (“OR”) criterion to FIBTEM. A combined FIBTEM and EXTEM A5 ≤ 35 mm approach was used by four hospitals (25%). ML thresholds varied, with ML >5% in five hospitals (31%) and ML ≥15% in one (6%). Fibrinolysis was also included as the final step in 10 (63%) hospitals, with ML >5% in four (25%) and ML ≥15% in six (38%). For ML specification, nine hospitals (56%) used EXTEM, four (25%) allowed either FIBTEM or EXTEM, and three (19%) mentioned ML without specifying the assay.

For hyperfibrinolysis management, all ML criteria recommended 1 g of TXA. One hospital (6%) used TXA alone for FIBTEM criteria, while 11 (69%) combined it with fibrinogen replacement, using fibrinogen concentrate (FC) (7, 44%) or both FC and cryoprecipitate (4, 25%). Fibrinogen dosing varied: 4 g (*n* = 7, 44%), 3 g (*n* = 2, 13%), or weight‐based 1 g/25 kg (*n* = 2, 13%). Among three hospitals (19%) that did not start their algorithm with hyperfibrinolysis, one (6%) administered TXA empirically for all critically bleeding patients, another (6%) combined empirical TXA with fibrinogen replacement for identified deficiency, and the third (6%) placed hyperfibrinolysis in the last step but included a note referring to it as the first step.

#### 3.1.2 Fibrinogen assessment

**FIGURE 2:**
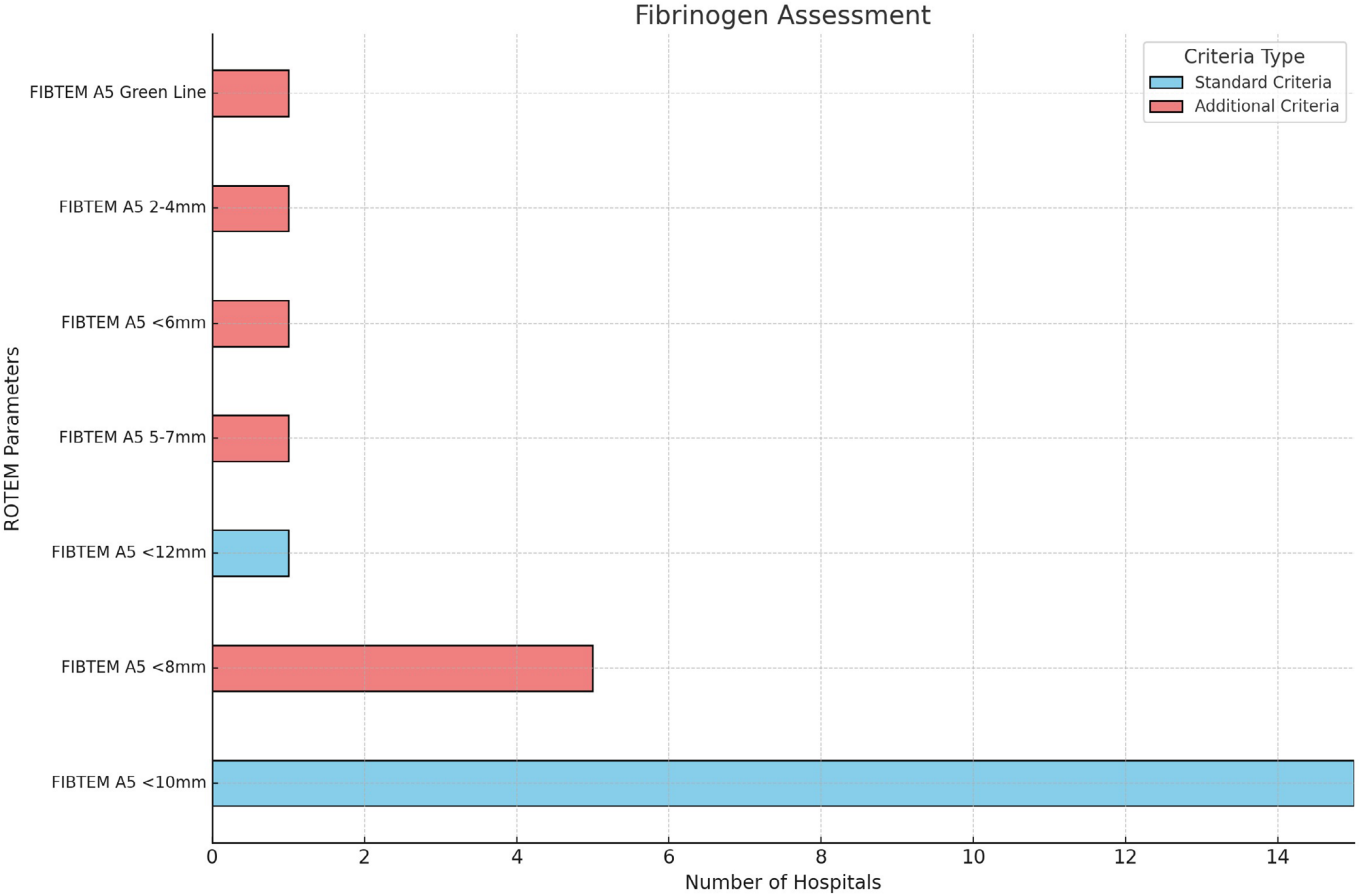
Threshold for fibrinogen replacement.

Out of 16 ROTEM algorithms, all included fibrinogen assessment using FIBTEM A5, primarily in the second step of clot assessment (*n* = 13, 81%) and occasionally as the first (*n* = 3, 19%). A threshold of FIBTEM A5 <10 mm was used in 15 hospitals (94%), while one (6%) used FIBTEM A5 <12 mm. FIBTEM A5 <8 mm was an additional criterion in 5 hospitals (31%) (Figure 2).

All algorithms recommended both cryoprecipitate and FC, with preferences varying by FIBTEM A5 threshold. FC was used exclusively in three algorithms (19%) for FIBTEM A5 <8 mm (*n* = 2) and FIBTEM Green Line (*n* = 1) while cryoprecipitate alone was recommended in eight (50%) for FIBTEM A5 <12 mm (*n* = 1), <10 mm (*n* = 4), 5–7 mm (*n* = 2), and 8–10 mm (*n* = 1). For other thresholds, both were offered as alternatives (“OR” option). One hospital recommended FC if cryoprecipitate was delayed >15 min with ongoing bleeding. Dosing for FIBTEM A5 <10 mm was 3–4 g FC or 10–20 units cryoprecipitate, with weight‐based options. For more severe deficiency, higher doses of both were recommended (Table 1).

#### 3.1.3 Platelet Assessment

**FIGURE 3:**
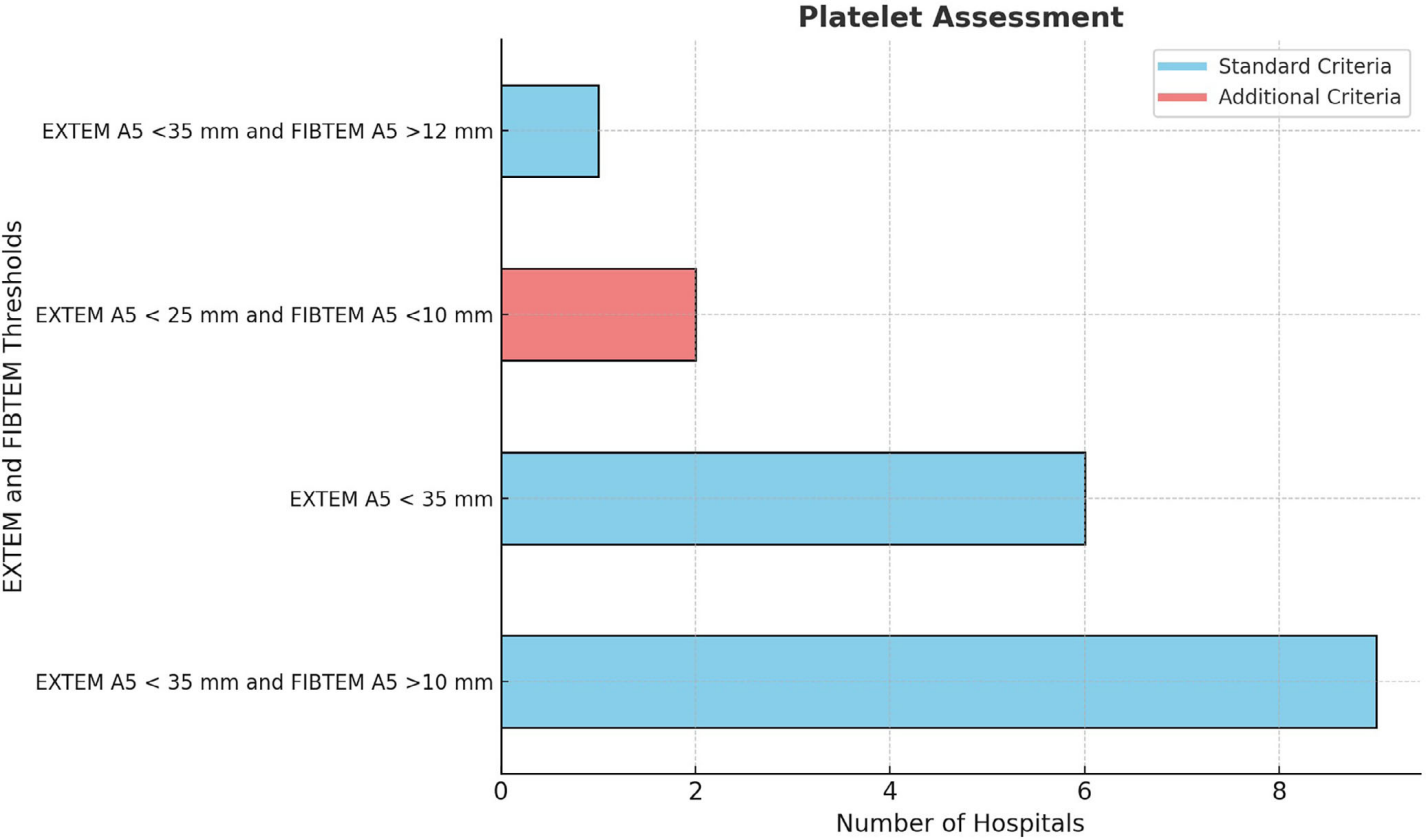
Thresholds for platelet replacement.

Out of 16 ROTEM algorithms, three (19%) used EXTEM A5 <35 mm alone, assuming FIBTEM A5 would be corrected to >10 mm, while nine (56%) explicitly mentioned FIBTEM A5 >10 mm as an “AND” condition with EXTEM A5 <35 mm (Figure 3). Two algorithms (13%) included EXTEM A5 <25 mm with FIBTEM A5 <10 mm as an additional platelet assessment criterion—one recommending platelet alone and the other recommending both platelets and fibrinogen, with a note to correlate with platelet count. Dosing was uniformly 1 unit of pooled platelets across all algorithms.

#### 3.1.4 Coagulation factor assessment

**FIGURE 4:**
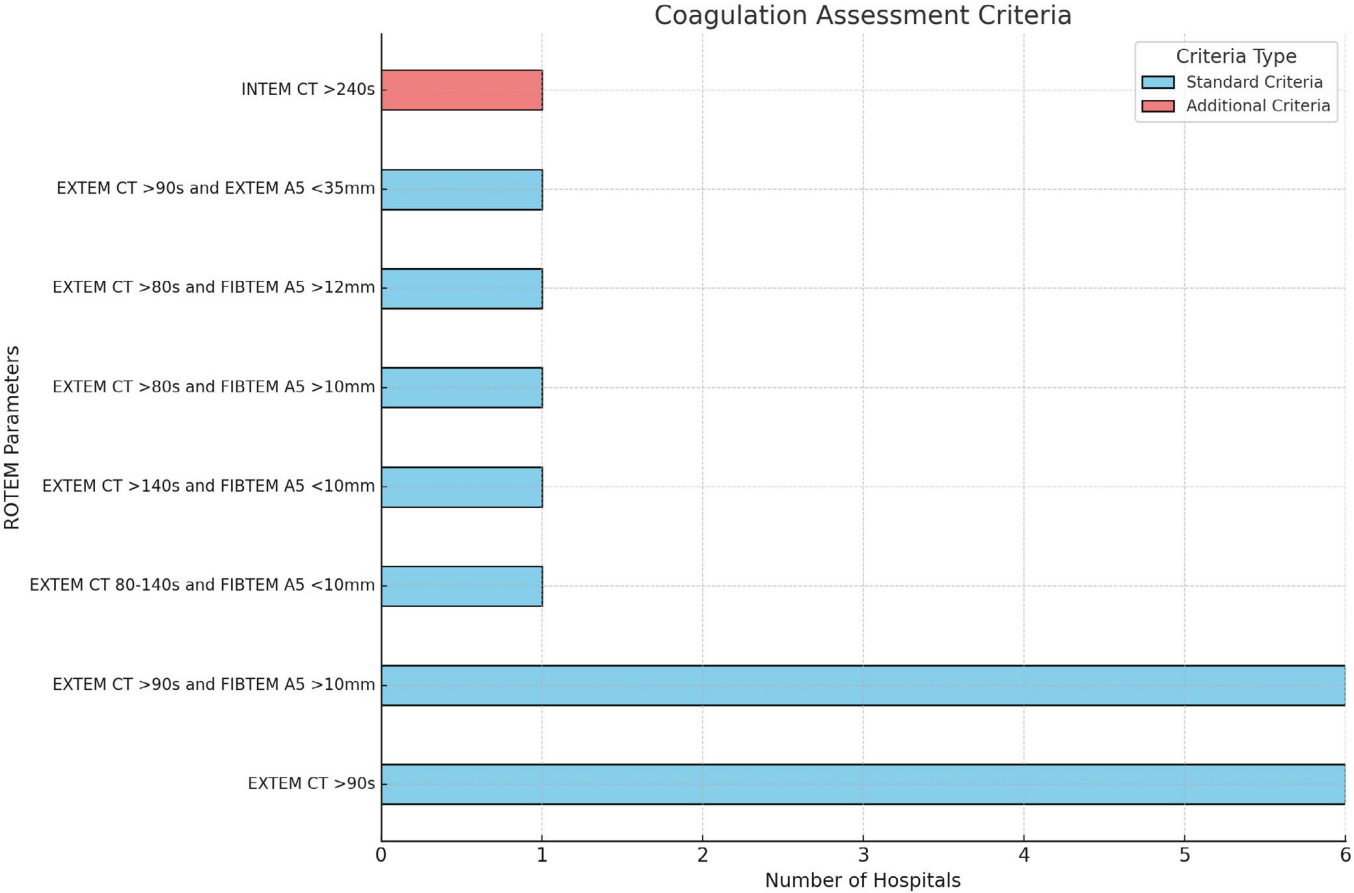
Thresholds for coagulation factor replacement.

Of 16 ROTEM algorithms, 12 (75%) used EXTEM CT >90 s, 2 (13%) used EXTEM CT >80 s, and 6 (38%) applied it standalone, assessing fibrinogen before coagulation factors. It was paired with FIBTEM A5 >10 mm in 5 (31%) and EXTEM A5 <35 mm in 1 (6%) (Figure 4). Additional criteria included EXTEM CT 80–140 s and >140 s (6%), INTEM CT ≥240 s (6%) and EXTEM CT 80–140 s and >140 s (*n* = 1). Five hospitals (31%) recommended fresh frozen plasma (FFP), one (6%) recommended prothrombin complex concentrate (PCC), and eight (50%) allowed either FFP or PCC. FFP dosing was consistently 2–4 units, while PCC dosing varied from 5 to 50 U/kg (Table 1). For EXTEM CT 80–140 s with FIBTEM A5 <10 mm, one hospital recommended fibrinogen, while for EXTEM CT >140 s with FIBTEM A5 <10 mm, FFP with fibrinogen replacement. None of the algorithms specify whether the recommended PCC refers to a 3‐ or 4‐factor formulation. While the algorithms were initially developed during a period when 3‐factor PCC was in use, they remain in practice following Queensland Health's transition to 4‐factor PCC. At the time of this manuscript write‐up, no formulation was explicitly stated in the protocols, and no dose modifications were noted in response to the transition.

**FIGURE 5:**
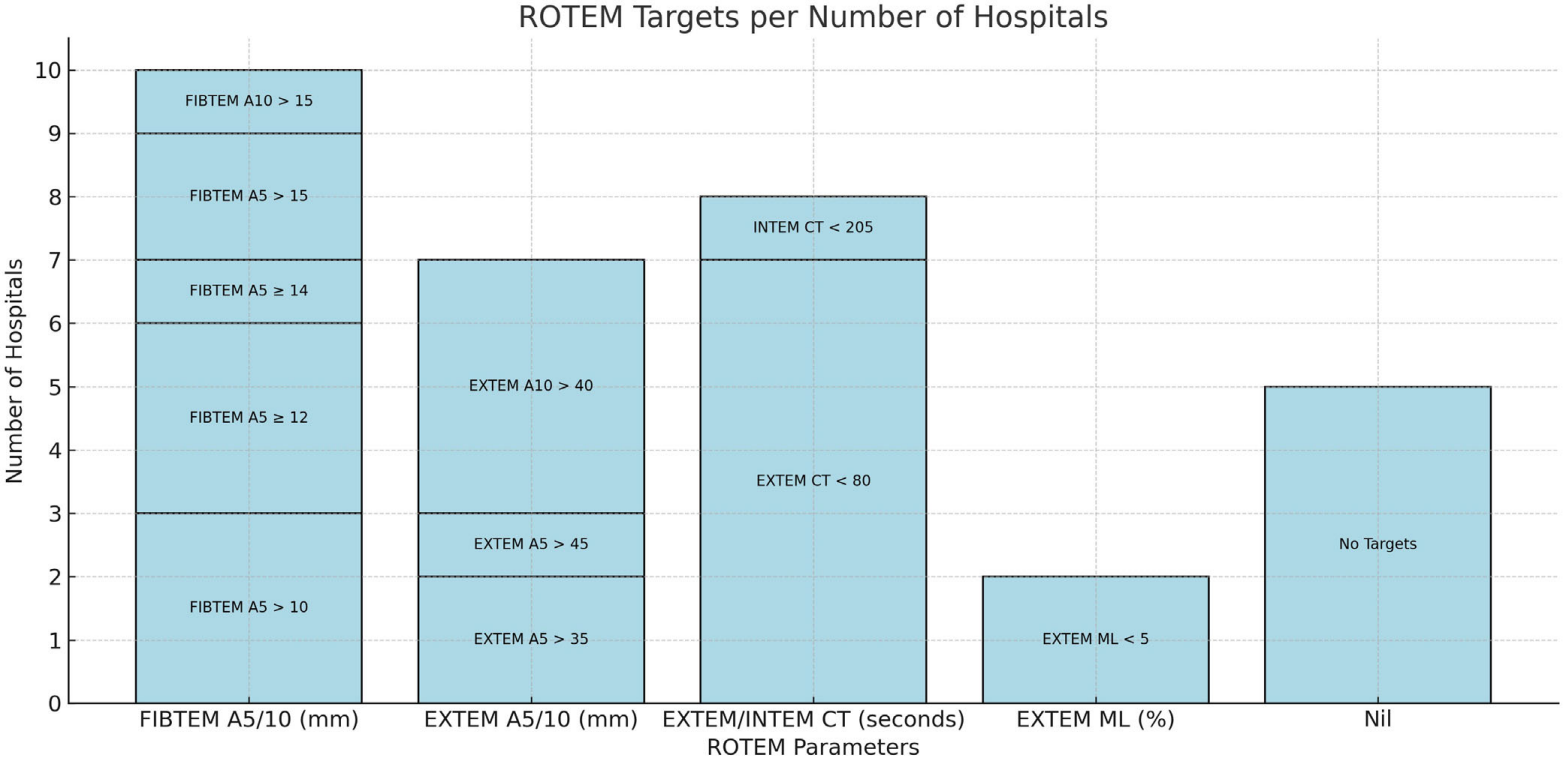
Recommended VHA‐based targets in MHPs.

Fifteen (94%) hospitals followed a stepwise haemostasis assessment: hyperfibrinolysis, fibrinogen, platelet, and coagulation factor. ROTEM‐based targets varied (Figure 5), with the most recommended parameters being FIBTEM A5/A10 (*n* = 10, 63%), EXTEM/INTEM CT (*n* = 8, 50%), EXTEM A5/A10 (*n* = 7, 44%), and EXTEM ML (*n* = 2, 13%). Five hospitals (31%) had no targets. The most consistent thresholds were EXTEM CT >80 s (*n* = 7, 44%), EXTEM A10 >40 mm (*n* = 4, 25%), and FIBTEM A5 >10 mm or ≥12 mm (*n* = 3, 31%). One hospital aimed for a normal ROTEM without specifying parameters. Among 13 hospitals (81%) with obstetric services, all targeted FIBTEM A5 >12 mm for obstetric bleeding.

### 3.2 TEG (n = 3)

Among hospitals which use TEG (*n* = 3), two hospitals followed a stepwise approach, prioritising fibrinogen, then platelets, coagulation factors, and hyperfibrinolysis. The third, without a stepwise approach, prioritised fibrinogen if CFF was abnormal, unless severe MA‐CRT deficiency was present, though severity was undefined. Two hospitals did not specify targets, while one aimed for a normal TEG.

All three TEG algorithms assessed fibrinogen using CFF A10 and/or CFF MA <15 mm, with one hospital using either, one relying on CFF MA, and another on CFF A10 (Table 1). One included more severe fibrinogen deficiency (CFF MA <10 mm and CFF MA <5 mm). Fibrinogen replacement varied: one recommended FC alone while two allowed FC or cryoprecipitate. Dosing varied: FC 2–3 g, cryoprecipitate 1 U/10 kg to 30 U (Table 1). For platelet assessment, criteria included CRT MA <50 mm (*n* = 2) and CRT A10 <44 mm (*n* = 1), with one hospital also using CRT MA <25 mm for severe deficiency. All recommended 1 unit of platelets, with one increasing to 2 for severe deficiency. Viscoelastic thresholds varied: CK R >9 min (*n* = 2) and CKH R >10 min (*n* = 1). All recommended FFP (2–4 U) or PCC (10–50 U/kg). Fibrinolysis criteria were consistent (LY30 >2.2%), with 1 g tranexamic acid. All hospitals assessed heparin effect (CKH R > CK R) and recommended 1 mg protamine per 100 U heparin.

## 4 DISCUSSION

This review of all hospital‐wide VHA algorithms in MHP across the statewide public health system in Queensland, Australia, identified variability in VHA indications, haemostatic thresholds, targets and transfusion recommendations. Although different algorithms have been published internationally for trauma, obstetrics, liver transplant, and cardiothoracic surgery, Queensland hospitals have selected a unifying algorithm when incorporating VHA into hospital‐wide MHPs. This standardised approach promotes consistent transfusion decision‐making, simplifies staff training, reduces variability in PBM, and streamlines blood product allocation.^8^, ^9^ Additionally, as some VHA devices are situated in blood banks, standardised hospital‐specific algorithms facilitate timely laboratory preparation and blood component release. However, a standardised algorithm may not adequately address distinct coagulopathy profiles within the same institution—for example, managing a variceal bleed in a patient with cirrhosis involves a markedly different haemostatic strategy than treating acute bleeding from a traumatic liver laceration, yet both may be subject to the same algorithm, potentially resulting in less individualised care.^10^, ^11^ Nationally, the National Blood Authority provides an MHP template for local adaptation, deferring VHA‐related decisions to site‐specific algorithms.^8^ This has led to a lack of national consensus and expected heterogeneity across jurisdictions. Internationally, MHPs do not delineate variability in VHA thresholds across different coagulopathic states and often favour population‐specific algorithms.^12^, ^13^, ^14^

Our study found that ROTEM was more commonly used than TEG and showed greater variation across all aspects of clot assessment, particularly in fibrinolysis timing (early vs. late) and assay selection, with FIBTEM CT used as a surrogate marker over ML for earlier diagnosis. TEG variations were less common but applied in fewer hospitals. For the same clot kinetics, dosing of blood products and haemostatic agents differed. While each hospital considers operational costs, logistical and blood availability, there is no statewide visibility into resources, expertise, training, or implementation considerations. Our findings indicate that patients presenting to different hospitals with the same haemostatic disturbance could be subjected to different viscoelastic thresholds and receive different haemostatic therapies. This study highlights the need to standardise VHA‐guided MHPs to minimise inter‐hospital variability and improve consistency, particularly for patients transferred between facilities.

Variability in fibrinolysis extends beyond timing of assessment and thresholds to how TXA is used in algorithms. Some hospitals recommend empiric TXA before ROTEM, while others administer it alongside fibrinogen replacement. FIBTEM‐based criteria typically combine TXA with fibrinogen replacement, while ML‐based criteria use TXA alone. FIBTEM CT values, available earlier than EXTEM ML, influence timing. Hyperfibrinolysis cut‐offs varied, with TEG algorithms consistent and ROTEM differentiating. Prior studies set maximal lysis >3% as critical in trauma or >15% as hyperfibrinolysis.^15^ Proposed ROTEM thresholds include EXTEM ML30 >15% or ML >20% for increased mortality and transfusion risk,^16^ EXTEM MCF <35 mm, ML >10% at 30 min or >15% at 60 min,^17^ and EXTEM/FIBTEM ML ≥5% within 60 min for hyperfibrinolysis in trauma.^18^, ^19^ In liver transplantation, A5 EX <25 mm and a flat‐line FIBTEM predicted fibrinolysis, with FIBTEM offering highest sensitivity by bypassing platelet‐mediated clot retraction.^19^, ^20^ Our findings align, with FIBTEM predominantly used for early and ML for late assessment. TEG algorithms consistently applied CRT LY30 >2.2%, addressing fibrinolysis last. A consensus panel advised against using VHA to withhold antifibrinolytics.^21^ Facilities with distant or infrequently used VHA machines should account for potential delays in VHA testing.

Discrepancies were observed between recommended blood products and their on‐site availability, particularly for platelets which were not stored on‐site in some hospitals despite being included in the algorithm or limited to one unit on‐site when two were recommended. This highlights the need to consider logistics and resources when developing VHA algorithms. FC was commonly recommended and prioritised for severe deficiencies or expected cryoprecipitate delays, likely influenced by Queensland studies showing faster replacement with FC.^22^ One hospital included an additional criterion for platelet assessment, recommending simultaneous fibrinogen and platelet replacement for severe clot firmness deficiency (EXTEM A5 <25 mm and FIBTEM A5 <10 mm), whereas other algorithms prioritised fibrinogen replacement first, followed by reassessment. This reflects differences in managing severe impairment in clot firmness.

Coagulation factor thresholds differed (EXTEM CT 80s, 90s, 80–140 s, >140 s; INTEM CT >240 s), reflecting the lack of a universal transfusion threshold and differences in diagnostic and treatment cut‐offs. EXTEM CT 75–91 s predicts INR elevation, coagulation factor deficiency, bleeding risk, transfusion needs, or mortality in surgery, liver transplant, and trauma.^21^, ^23^, ^24^, ^25^, ^26^, ^27^, ^28^, ^29^, ^30^, ^31^ INTEM CT >240 s guided factor replacement if EXTEM CT >80s and FIBTEM A5 >10 mm were unmet, while liver algorithms used 280 s.^2^ FFP dosing remained 2–4 units, while PCC varied widely (5–50 IU/kg). Australia now uses 4‐factor PCC (Beriplex AU) for acquired prothrombin complex deficiencies related to vitamin K antagonists, capping doses at 100 kg.^32^ As a lyophilized product, PCC is of immediate availability, requires lower volumes, and generates more thrombin than plasma, with one Beriplex 500 vial (~500 IU thrombin, factor II) equalling ~2.5 FFP units.^32^, ^33^ An RCT found empiric 4F‐PCC in trauma increased thromboembolic events without reducing 24‐h blood use.^34^ However, in goal‐directed VHA, a favourable survival rate was observed.^35^ PCC normalised EXTEM CT <80s in trauma.^36^ Previous studies show marked variation in PCC dosing in VHA.^36^, ^37^ The optimal dose remains unclear; protocols should now incorporate a dose cap at 100 kg.

This review focused on VHA algorithms in hospital‐wide MHPs, ensuring comparability. Specialised areas, such as cardiothoracic and liver transplant, use distinct algorithms which were not assessed. It does not account for hospital type (trauma centres, cardiac, tertiary, regional facilities, obstetric‐focused hospitals), nor demographic variation in coagulopathy. TEG use was limited to a few public hospitals, likely leading to underreporting of variations. There are no statewide notifications for algorithm updates; new changes may not have been captured, though all algorithms were current at review. The study did not evaluate hospital resources, surgical capabilities, blood delivery timing, or multidisciplinary challenges of MHP. Developing VHA algorithms requires multidisciplinary collaboration and capturing challenges was beyond the scope of this review.

## 5 CONCLUSION

Substantial variation in VHA‐guided algorithms was observed statewide, with inconsistent transfusion thresholds. For the same clot kinetics, dosing of blood products and haemostatic agents differed, highlighting the need for standardisation. Patients with the same type and degree of haemostatic disturbance face varying haemostatic management across various hospitals within the same statewide health service. While FC and PCC were commonly recommended, dosing of PCC varied markedly. A centralised effort to harmonise algorithms, supported by education and logistics, could improve PBM consistency for bleeding.

## AUTHOR CONTRIBUTIONS

Akmez Latona designed the research study, collected and analysed the data, performed the research and wrote the manuscript. James Winearls, Kate Hill, Michelle Spanevello, Biswadev Mitra edited the manuscript.

## FUNDING INFORMATION

Although not directly funding this work, Dr. Akmez Latona acknowledges funding from the Emergency Medicine Foundation (grant ID 233R38‐2022‐LATONA). This grant supported and contributed to the environment that stimulated this work.

## CONFLICT OF INTEREST STATEMENT

There are no actual or perceived conflicts of interest (from all authors) in the conduct or reporting of the research.

## Data Availability

Data sharing is not applicable to this article as no datasets were generated or analysed.
